# Atypical Case of COVID-19 Associated Kawasaki Disease in an Eight-Year-Old Pakistani Boy

**DOI:** 10.7759/cureus.10670

**Published:** 2020-09-26

**Authors:** Israr Khan, Ayesha Sarwar, Zahoor Ahmed

**Affiliations:** 1 Internal Medicine, Bolan Medical College, Quetta, PAK; 2 Internal Medicine, King Edward Medical University, Mayo Hospital, Lahore, PAK

**Keywords:** covid-19, sars-cov-2, kawasaki disease, vasculitis, mucocutaneous lymph node syndrome

## Abstract

People of all age groups have been affected worldwide during the coronavirus disease 2019 (COVID-19) pandemic. While the global efforts of researchers, clinicians, and scientists are underway, cases involving multiple systems with a wide range of presentations are on the horizon. As health organizations have started warnings about unusual manifestations of a Kawasaki disease (KD)-like inflammatory syndrome associated with COVID-19, some pediatric cardiologists noted that even classic cases are likely going undercounted. Here we report a case of a previously healthy eight-year-old Pakistani boy who presented with a four-day history of low-grade fever. The patient was admitted and diagnosed with COVID-19-associated atypical KD in the setting of fever for more than five days, maculopapular eruptions, and mild conjunctivitis. He screened positive for COVID-19 with an immunoglobulin G titer of 2.1 plus ruling out other childhood illnesses. He was managed with intravenous immunoglobulins and aspirin with gradual resolution of symptoms. His initial echocardiogram was unremarkable. He was discharged home on day six with a follow-up at two weeks.

## Introduction

Coronavirus disease 2019 (COVID-19) has affected people of all age groups to some extent. However, only 2% of its cases have been reported in individuals younger than 20 years. During this pandemic, a relatively rare form of vasculitis known as Kawasaki disease (KD), which affects medium-sized blood vessels, has started surfacing worldwide in post-COVID-19 children. KD is an acute, self-resolving vasculitis, with a slight male to female predominance of 1.5:1 [1]. It was first reported in 1967 by Tomisaku Kawasaki in Japan, and it has replaced acute rheumatic fever as the leading cause of acquired heart disease among children around the globe, which requires a periodic cardiovascular assessment of post-KD children [2]. Since there is no diagnostic test to diagnose KD, these children are usually diagnosed according to international criteria after thoroughly considering their clinical presentation and exclusion of other possible causes, (e.g., Staphylococcal Scalded Skin Syndrome [SSSS], Steven-Johnson Syndrome, Streptococcal scarlet fever, viral infection, and drug allergy) [3].

## Case presentation

An eight-year-old boy without significant past medical history was brought to the outpatient department by his parents with a four-day history of non-documented, low-grade intermittent fever, which became persistent from the very next day. There were no associated symptoms at the time of the presentation. Additionally, parents reported no history of sore throat, cough, nausea, vomiting, and bowel and bladder abnormalities. There was no history of any drug intake, contact with a sick patient, or known exposure to someone COVID-19 positive. Initial evaluation revealed a temperature of 99°F, blood pressure (BP) of 115/75 mmHg, heart rate (HR) of 90 beats/minute, respiratory rate (RR) of 18 breaths/minute, and oxygen saturation (SpO_2_) of 98% while breathing ambient air.

The patient was discharged on acetaminophen (Tylenol). The next day, the patient presented with a fever again, but this fever was accompanied by lethargy and tachypnea. He also developed mild-to-moderate maculopapular eruptions all over the body with sandpaper-like texture and a mild degree of bilateral conjunctivitis.

**Figure 1:**
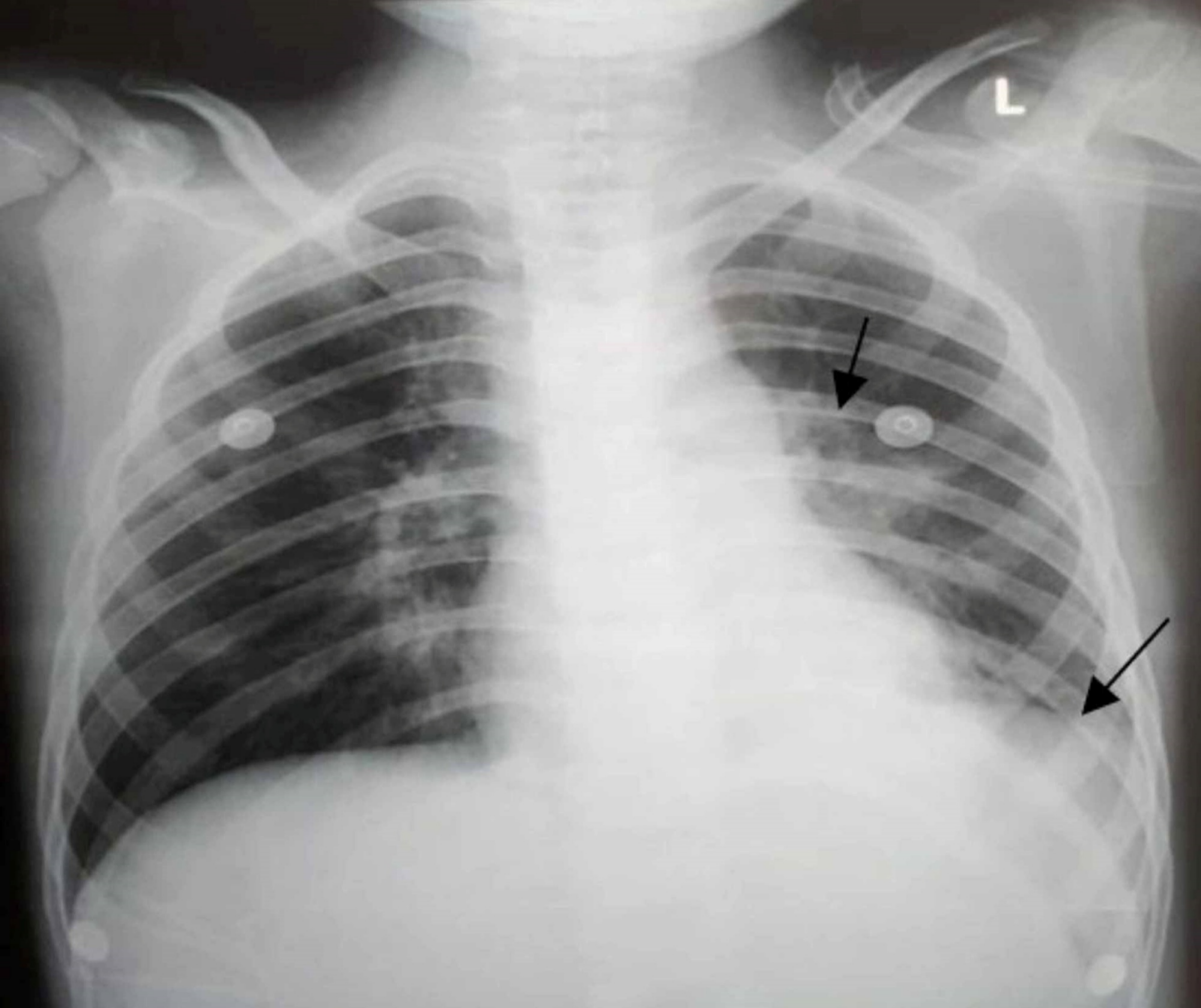
Chest x-ray posteroanterior view showing left upper lobe dense area of parenchymal opacification in addition to pleural effusion involving left lower lobe

He was admitted to the pediatric floor, and per hospital protocol, reverse transcriptase-polymerase chain reaction for COVID-19 was also advised along with other laboratory investigations. Physical examination at the time of the second presentation demonstrated a temperature of 100°F, BP of 105/75 mmHg, HR of 95 beats/minute, RR of 27 breaths/minute, and SpO_2_ of 91%. His workup revealed the following: white blood cell count of 10 x 109/L, a prothrombin time of 19.3 seconds, an activated partial thromboplastin time of 33.2 seconds, C-reactive protein (CRP) of 41 mg/L, an erythrocyte sedimentation rate (ESR) of 30, and a D-dimer concentration of 1.8 µg/ml. A chest x-ray (CXR) showed a parenchymal opacification in the left upper lobe with pleural effusion on the same side (Figure 1). His COVID-19 test result was negative.

**Figure 2:**
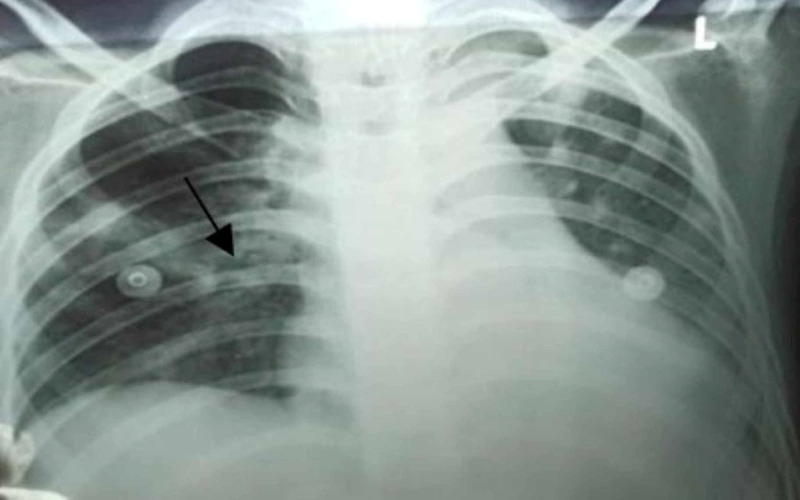
Chest x-ray posteroanterior view showing patchy infiltrates in the right middle lobe

The patient was empirically started on 1.2 g of intravenous (IV) ceftriaxone and 320 mg of IV vancomycin three times per day in normal saline at a slow rate. In addition, echocardiography was performed. On the following day, his fever was 101°F, and his SpO_2_ started dropping dramatically to 85% along with worsening tachypnea. He was started on supplemental oxygen at 2 L/min via face mask. A portable CXR was done, which showed worsened inflammatory changes with local patchy infiltrates in the right middle lobe (Figure 2).

The echocardiography findings were unremarkable. Blood serology for measles was negative. Additionally, a strep throat test result was negative. After carefully ruling out all possible differentials like a drug-induced allergic reaction and infections (e.g., SSSS, scarlet fever, measles, varicella), the patient was highly suspected of having atypical KD based on the clinical presentation and history of fever. He was also started on high-dose IV immunoglobulins (IVIG) at 2 g/kg on day one (given the patient weighed 24 kg) along with a high dose of aspirin (80 mg/kg/day every six hours) as per guidelines. Moreover, an anti-severe acute respiratory syndrome-coronavirus-2 serologic test result was positive, with an immunoglobulin G titer level of 2.1. Hence, he was diagnosed with COVID-19-associated atypical KD.

**Figure 3:**
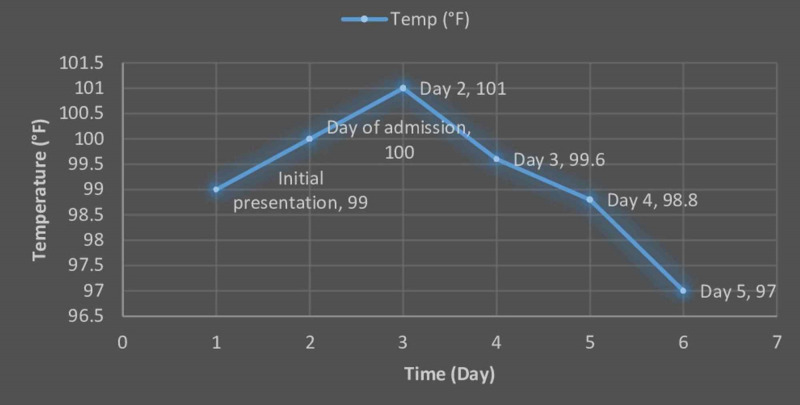
Daily temperature graph of the patient during hospital stay

Twenty-four hours after completion of IVIG, his breathing issues started resolving. His repeated CXR showed mild inflammatory changes without any new lung infiltrates or lesions. Additionally, his fever also subsided, and the rash started disappearing. His daily temperature chart is shown in Figure 3. On day six, he was discharged from the hospital on a low dose aspirin of (5 mg/kg/day) as maintenance therapy. On his follow-up visit, the patient was doing well.

## Discussion

An association between COVID-19 and KD has been reported in several case reports [4]. An observational study by Verdoni et al. also reported an outbreak of severe Kawasaki-like disease at the Italian epicenter during the epidemic [5]. The severity of the disease in infants and children with COVID-19 is far less terrible than that of adults. In the USA, children younger than 18 years have comprised only 1.7% of the total cases [6].

KD is a disease of children affecting medium-sized arteries throughout the body. KD has a wide range of signs and symptoms. The patient usually presents with high-grade fever, rash, red eyes, swollen glands, and strawberry tongue. In addition to these signs and symptoms, children can also present with gastrointestinal symptoms and joint pain [7]. The diagnosis of KD is based on clinical, serological, and imaging modalities. To diagnose, a patient must have four of the following symptoms according to Ouldali et al.: “bilateral conjunctival injection, changes affecting the lips and oral cavity (inflammation of the oral and pharyngeal mucosa), polymorphic exanthema, changes in the peripheral extremities or perineal area, and cervical lymphadenopathy of 15 mm or larger” [8]. Serology includes blood tests, ESR, and CRP. Imaging modalities include x-ray, computed tomography, and heart studies like echocardiography and coronary angiogram. Children with persistent fever (i.e., lasting five days or longer) with two to three principal criteria of typical KD should be evaluated for incomplete KD. The presence of a positive echocardiogram, (i.e., a Z-score of the left anterior descending coronary artery or right coronary artery ≥ 2.5 mm or coronary artery aneurysm) confirms the diagnosis.

Management of KD depends upon the clinical presentation and severity of the disease. The most recommended therapy is IVIG. Aspirin and blood thinner drugs can be prescribed based on the patient’s condition [6]. In severe cases, the child might have surgery. Infants have a high risk of heart complications like arrhythmia, myocarditis, and valvular regurgitation.

## Conclusions

Our case report highlights an atypical manifestation of KD due to COVID-19. This case particularly emphasizes the significance of clinical examination and a high index of suspicion for atypical KD in children during this pandemic. This case also highlights the importance of early investigation and early implementation IVIG in a patient with Kawasaki disease. While both classical and atypical cases of KD are on the rise, at present many cases go undetected with a similar presentation of other diseases and COVID-19 in particular. Children with symptoms like fever lasting five days or longer, erythematous rash, and conjunctivitis should be swiftly evaluated by pediatric cardiologists, especially when other childhood illnesses are thoroughly ruled out, to ensure early diagnosis and prompt management of KD to reduce the morbidity and mortality.
